# MMP-9 Levels in the Gingival Crevicular Fluid of Chilean Rosacea Patients

**DOI:** 10.3390/ijms23179858

**Published:** 2022-08-30

**Authors:** Javier Fernández, Constanza Jiménez, Dafna Benadof, Paulina Morales, Jessica Astorga, Felipe Cáceres, Marcela Hernández, Alejandra Fernández, Fernando Valenzuela

**Affiliations:** 1 Centro Internacional de Estudios Clínicos, Probity Medical Research, Santiago 8420383, Chile; 2 Department of Oral Pathology, Faculty of Dentistry, Universidad Andres Bello, Santiago 8370133, Chile; 3 Laboratory of Periodontal Biology, Faculty of Dentistry, Universidad de Chile, Santiago 8380544, Chile; 4 Department of Oral Pathology and Medicine, Faculty of Dentistry, Universidad de Chile, Santiago 8380544, Chile; 5 Department of Dermatology, Faculty of Medicine, Universidad de Los Andes, Av. Plaza 2501, Las Condes, Santiago 7620157, Chile

**Keywords:** biomarkers, rosacea, gingival crevicular fluid

## Abstract

Rosacea is a chronic inflammatory skin disease whose prevalence rates remain unknown in Chile. Laboratory benchmark testing for this disease is not useful, therefore, we aimed to evaluate the gingival crevicular fluid (GCF) levels of extracellular metalloproteinases (MMP)-2 and MMP-9 as novel rosacea biomarkers. We designed a cross-sectional study with a control group. Participants were systemically healthy adults (*n* = 20) and persons with rosacea (*n* = 18). We performed a periodontal evaluation and collected gingival crevicular fluid to measure MMP-2 and MMP-9 levels. Analysis showed mean and standard deviation of MMP-9 concentrations in the GCF for patients with rosacea was 764.52 ± 569.83 pg/mL; for healthy patients, it was 260.69 ± 170.43 pg/mL (*p* < 0.05). The diagnosis of rosacea was responsible for the levels of MMP-9 in the GCF (*p* < 0.05), as opposed to periodontitis, smoking, and age (*p* > 0.05). The Area under ROC for MMP-9 was 0.869 (95%, C.I: 0.719–0.956), with a sensitivity of 72.22% and specificity of 81.58% for the diagnosis of rosacea. We conclude that the quantification of MMP-9 in the GCF could be used as a biomarker of rosacea. Also, rosacea was responsible for increasing the levels of MMP-9 in the GCF independent of periodontal status.

## 1. Introduction

Rosacea is a chronic inflammatory skin disease that manifests in highly visible areas of the body, including the skin of the face. Dermatosis affects 5.46% of the worldwide population [1] with no apparent gender preference [2] and an increasing incidence with age. In general, rosacea is more frequently seen in fair-skinned middle-aged women primarily because they consult more with their healthcare providers) and less frequently in men and skin phototypes V and VI [3]. To this date, the exact prevalence rates for rosacea in Chile remain unknown.

Although rosacea is clinically diagnosed based on a compatible medical history and physical examination, its identification may be challenging for physicians due to the frequent overlap with chronic actinic damage [4]. Laboratory benchmark testing for rosacea is not useful; hence misdiagnosis with other conditions such as adult acne vulgaris, atopic dermatitis, and seborrheic dermatitis is common [5]. Primary features of rosacea include the presence of at least one of the following symptoms: diffuse non-transient central facial erythema, telangiectasia, papules/pustules, and flushing [6]. Secondary features comprise burning or stinging, edema, a dry skin appearance, plaque formation, and lesions in peripheral locations. Ocular involvement and rhinophyma (phymatous changes on the skin of the nose that cause permanent disfiguration) are also identified in severe cases [7]. In addition, patients with rosacea describe episodes of clinical intensification of symptoms associated with external triggering factors such as sun exposure, heat/cold, and the consumption of spicy foods or alcohol, hinting at a complex and multifactorial pathophysiology and a long-term clinical course characterized by relapses [8].

Because facial skin participates in social interactions, patients with rosacea usually report a significant disease burden in their daily, social, and professional lives. Feelings of social stigmatization [9], shame, low self-esteem, anxiety, and depression [10] are frequent in rosacea patients, leading to a substantial decline in their overall well-being and quality of life [10]. In addition, the recent association of rosacea with other non-communicable chronic diseases such as inflammatory bowel disease [11], cardiovascular disease [12], insulin resistance [13], and mental disorders [14] has changed the perception of rosacea from a “local skin condition” to chronic systemic disease [8].

The exact biological and molecular mechanisms behind rosacea’s pathogenesis are poorly understood; nonetheless, dysregulation of the neurovascular and immune systems is highly probable [6]. Alterations in the innate immune response and angiogenesis of the skin, mediated by the Toll-like receptor (TLR) 2/NF-kB signaling pathway, seem to play an essential role in the pathogenesis of the disease [15,16,17,18]. The involvement of TLR-2 receptors in rosacea responds to diverse stimuli such as the production of reactive oxygen species, solar radiation, microbial dysbiosis (*Staphylococcus epidermidis*), and the presence of microbial proteins and other byproducts [19]. Activation of the TLR-2/NF-kB pathway stimulates the production and secretion of pro-inflammatory cytokines in skin keratinocytes, favoring the establishment of a chronic inflammatory state in the dermis and epidermis of rosacea patients [20]. In addition, antimicrobial peptides such as LL-37 may also activate the TLR-2/NF-kB pathway in the disease [17]. LL-37 is the active form of cathelicidin, cleaved by kallikrein 5 (KLK5), and is expressed on neutrophils, macrophages, and mast cells [21]. KLK5, on the other hand, is activated by extracellular metalloproteinases (MMPs) from its proenzyme [6]. LL-37 levels are higher in facial skin samples of rosacea patients compared to systemically healthy controls, hinting at its participation in rosacea’s pathogenesis [22]. Experimental laboratory models have shown that intradermal injections of LL-37 increase the expression of TNF-alpha, interleukin (IL)-6, vascular endothelial growth factor (VEGF), and MMP-9 in the skin of mice with rosacea compared to controls [17], indicating that these proteins are involved in the pathogenesis of the dermatosis.

MMPs are zinc-dependent endopeptidases that degrade different extracellular matrix constituents in chronic skin diseases [23]. Dermal keratinocytes and fibroblasts synthesize MMPs in response to chronic exposure to several stimuli such as ultraviolet radiation and pro-inflammatory cytokines [24,25]. Among the different types of MMPs, MMP-2 and -9 have been implicated as participants in the pathogenesis of rosacea [26,27]. These MMPs belong to the gelatinase family and cooperatively participate in the degradation of gelatin and collagen type I [28]. Few studies have assessed the expression of MMP-9 in facial skin samples of rosacea patients [21,29]; nonetheless, several studies have explored its levels in other bodily fluids such as tears and serum [30,31]. Interestingly, results from those studies indicate that rosacea has the potential to modify the concentrations of MMP-9 in these bodily fluids at a distance, hinting at a broader and “systemic” impact of the disease. These findings align with results previously published by our research group, where we found that psoriasis and atopic dermatitis can influence the composition of the GCF [7,32,33]. Therefore, detecting biomarkers in human bodily fluids can help determine whether rosacea can affect changes in distant tissues.

Biomarkers are molecules that can be objectively measured in biological fluids (i.e., serum, saliva, gingival crevicular fluid, etc.). Alterations in their concentrations signal abnormal processes, conditions, or diseases [34]. In a previous study, we successfully associated gingival crevicular fluid metalloproteinases (MMPs) with atopic dermatitis regardless of periodontal status [7]. The search for gingival crevicular fluid biomarkers for the early diagnosis of systemic diseases has the advantage of being a safe, non-invasive, and comparatively easy-to-perform technique, which offers one of the most readily accessible entrees to any of the tissues in the human body [34,35]. To this day, the diagnosis of rosacea remains a primarily clinical process, based on comprehensive history-taking and the observation of skin lesions and clinical symptoms [5]. The presence of centrofacial erythema, flushing, telangiectasia, and papules/pustules is critical [28]. Skin biopsies are usually unnecessary; nevertheless, they might still be crucial for differential diagnosis with other similar yet pernicious conditions such as angiosarcoma [29]. Therefore, it is necessary to have certainty in the early diagnosis of rosacea and complement it with the detection of specific biomarkers of this disease [30]. In this study, we hypothesize that rosacea influences the GCF levels of MMP-2 and -9 regardless of periodontal status and thus can be used as a novel diagnostic biomarker for rosacea. This manuscript aims to compare the GCF levels of MMP-2 and MMP-9 in systemically healthy and rosacea patients and to evaluate their potential use as novel disease biomarkers.

## 2. Results

The samples of the GCF were obtained from 38 individuals, of which 18 presented the diagnosis of rosacea, and 20 corresponded to systemically healthy patients. The results of the demographic and clinical periodontal parameters are shown in Table 1. The table shows no significant differences in the average age, probing depth, clinical attachment level, bleeding on probing index, the percentage of smokers, and the presence of periodontitis (*p* > 0.05). However, there was a difference between the percentage of women and men included in the study (*p* < 0.05). Specifically, the rosacea group was constituted mainly of females (94.44%) versus 5.56% of males. Similarly, the control group comprised primarily of females (65%) versus 35% males.

**Figure 1:**
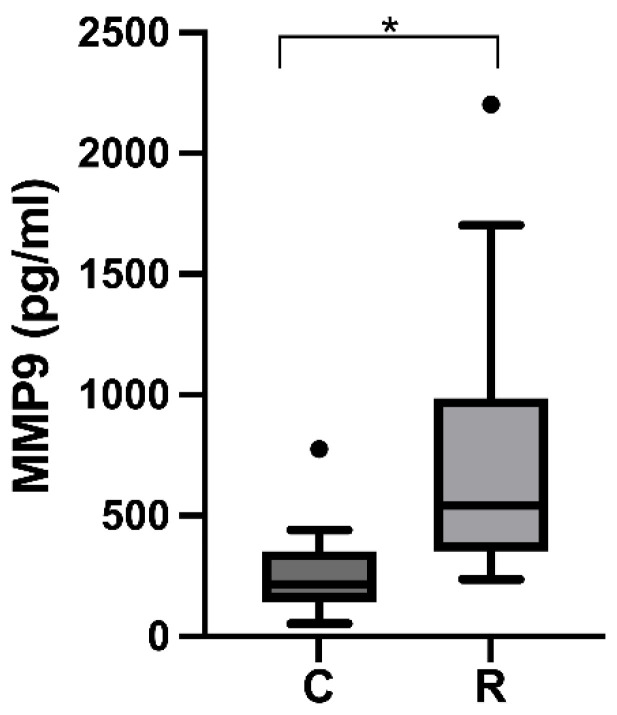
MMP-9 levels in the gingival crevicular fluid in rosacea patients and controls. Higher levels of mmp-9 were observed in the GCF of rosacea patients compared to controls, * *p* < 0.0001. C: control, R: rosacea. Black dot = outlier.

The results of MMP-9 levels in the GCF of patients with rosacea and healthy controls are shown in Table 2. The mean and standard deviation of MMP-9 concentrations in the GCF for patients with rosacea was 764.52 ± 569.83 pg/mL; for healthy patients, it was 260.69 ± 170.43 pg/mL. Therefore, rosacea patients presented higher levels of MMP-9 in the GCF compared to healthy control patients (*p* < 0.05), Figure 1. Also, we could not quantify the levels of MMP-2 in the GCF of rosacea patients and healthy subjects due to MMP-2 concentrations being below the range detection limit (86.6 pg/mL) awarded by the kit.

Then, to know if the upregulation of MMP-9 in the GCF of patients with rosacea is due to this disease, a multiple regression model adjusted for periodontitis, smoking, and age was performed. Table 2 shows that the diagnosis of rosacea is responsible for the levels of MMP-9 in the GCF (*p* < 0.05) and not periodontitis, smoking, and age (*p* > 0.05).

**Figure 2:**
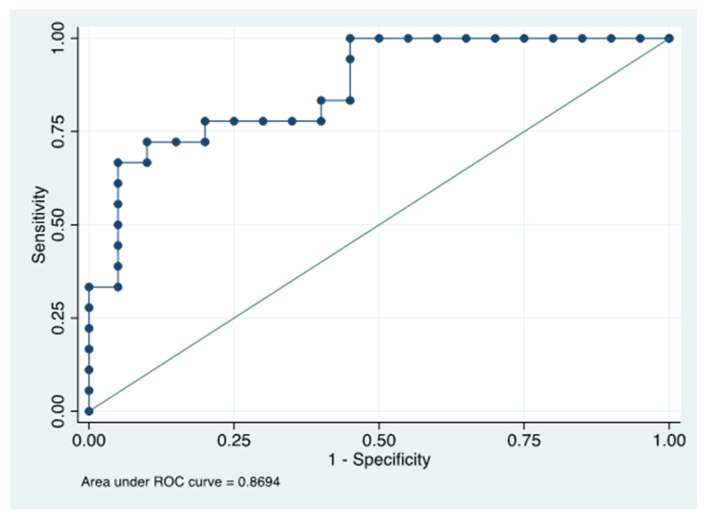
Receiver operating characteristic (ROC) curves for MMP-9 to evaluate its biomarker’s ability. The line drawn from the 0.00 to the 1.00 point corresponds to the reference diagonal.

Finally, we analyzed the ROC curve (receiver operating characteristic curve) for MMP-9 in the GCF to determine the diagnostic accuracy of rosacea. The ROC curve for MMP-9 is exhibited in Figure 2. The Area under ROC for MMP-9 was 0.869 (95%, C.I: 0.719–0.956), with a sensitivity of 72.22% and specificity of 90.5% for the diagnosis of rosacea.

## 3. Discussion

Rosacea is a chronic inflammatory disease currently proposed to be a systemic pathology [8]. The etiopathogenesis of rosacea is not clear. However, it is known that deregulation of the immune system culminates in the synthesis of antimicrobial peptides, proinflammatory cytokines, and MMPs, which favor a local inflammatory environment and vascular changes in the skin [7,31]. Interestingly, several studies have reported the upregulation of interleukins and MMPs, in samples collected from tear and serum to be associated with the pathogenesis of rosacea [7,32,33,34,35]. Therefore, according to these studies, rosacea could cause changes in the composition of proteins in bodily fluids. Our group also reported that psoriasis and atopic dermatitis could influence the GCF’s concentrations of cytokines and MMP, respectively [25,36]. Since rosacea shares specific inflammation mechanisms with psoriasis and atopic dermatitis [37,38], we hypothesized that rosacea might also modify the protein composition in the GCF. To date, there is no evidence of this relationship, let alone the role, of MMP-9 in the GCF in individuals with rosacea. In the present study, we report higher levels of MMP-9 in the GCF of patients with rosacea compared to systemically healthy controls. This phenomenon was attributed to the presence of rosacea and not necessarily to the periodontal status of the individual. Also, these results indicate that rosacea could also produce changes in the composition of the GCF, enhancing rosacea as a systemic disease. Finally, the quantification of MMP-9 in the GCF could be used as a biomarker of rosacea.

In rosacea, when LL-37 is present, mast cells are partially responsible for changing the expression and activity of MMP-9 [16]. Keratinocytes also increase MMP-9 secretion in a dose-dependent ultraviolet B radiation [39] and a pro-inflammatory environment [40]. Some studies have evaluated the expression of MMP-9 in rosacea [16,41]; however its specific role is ascertained in this disease. Higher levels of MMP-9 mRNA have been detected in facial skin samples from rosacea patients compared to the facial skin of healthy subjects, suggesting that MMP-9 is involved in the development of rosacea [16]. In addition, the immunoexpression of MMP-9 in the dermis of rosacea Granulomatosa biopsies (advanced stage of rosacea) was higher than in rosacea non-Granulomatosa [41], suggesting that MMP-9 participates in the progression of rosacea. Despite these two studies available in the literature, more studies are needed to determine the role of MMP-9 at the local level in rosacea. Dermal pathologists have mainly studied the function of MMP-9 in chronic wounds and psoriasis [42,43]. In a murine wound model, they showed that the presence of active exogenous MMP-9 produced a delay in the closure of the lesion [42]. This may indicate that active MMP-9 constantly degrades the basement membrane between the epidermis and dermis, making it difficult for keratinocytes to adhere and heal [42]. The activity of MMP-9 in the skin could be essential to define its role in physiological and pathological situations. One study showed that MMP-9 activates endothelial cells, facilitating the transmigration of CD4+ T cells in vitro. Additionally, the same study reported that MMP-9 inhibition decreases vascular permeability and cutaneous vasodilation in an animal model of psoriasis [43]. Therefore, MMP-9 may be responsible for important features of rosacea, such as the development of permanent erythema and facial telangiectasias and the migration of inflammatory cells to affected facial skin.

Rosacea presents extra-facial involvement, for which biomarkers have been found in tear fluid and serum associated with cutaneous inflammation [32,44]. The ocular manifestations of rosacea mainly include papulopustular eruptions on a telangiectatic background. These manifestations may occur before dermal ones on the face [45]. Higher levels of MMP-9 have been detected in tear fluid and serum, as well as pro-MMP9 in tears of patients with rosacea, compared to healthy patients, respectively [24,34,46]. These results suggest that rosacea may cause changes in MMP-9 at a distance. Tears of rosacea patients decreased after doxycycline treatment, suggesting the potential use of MMP-9 as a therapeutic biomarker for rosacea [34]. It increases the expression of MMP in human keratinocytes and cell lines [47,48], and its use is indicated in the treatment of rosacea. In our study, we also found higher MMP-9 levels in the GCF of individuals with rosacea versus healthy controls, confirming that rosacea induces changes in biomarkers associated with the inflammation of this disease at a distance from the facial skin. Although higher levels of MMP-9 were reported in atopic dermatitis skin wash samples compared to normal controls [49], our investigation group did not detect a difference in the MMP-9 levels in GCF between Atopic Dermatitis patients and healthy controls [25]. Therefore, we suggest that the MMP-9 present in the facial skin of individuals with rosacea enters the circulation due to increased vascular permeability (provided by MMP-9 itself), driving it to the periodontal tissues.

At the periodontal level, MMP-9 is mainly synthesized by neutrophils in the presence of inflammation [50], and a higher number of oral neutrophils were detected in periodontitis patients in relation to healthy control [51]. MMP-9 is one of the main MMPs associated with periodontal bone destruction [52]. Unlike the evaluation of rosacea, the levels of MMP-9 at the periodontal and systemic levels have been widely evaluated. Studies have detected higher levels of MMP-9 in the GCF and serum of individuals with periodontitis compared to healthy individuals [53,54]. Increased MMP-9 activity has been associated with increased probing depth (a parameter that measures epithelial attachment loss) [54], which may reflect that at a higher MMP-9 activity in the GCF, there is greater destruction of the periodontal tissue. In the present study, we did not find a difference in mean probing depth in the rosacea group compared to the control group. Therefore, it is plausible that the elevated MMP-9 levels in the GCF of rosacea individuals cannot be explained by periodontal destruction. In addition, the presence of neutrophils in the periodontal tissue of rosacea patients has not been evaluated. Still, it has been published that neutrophils in rosacea can exert anti-inflammatory effects [55]. Therefore, we suggest that mast cells and keratinocytes in patients’ skin increase the secretion of MMP-9, which could arrive GCF by circulation.

Even though MMPs have historically been associated with the degradation of ECM in soft and hard tissues, MMP-9 also seems to play a protective role in the inflammatory response and osteolytic processes in periodontal tissue [56,57]. Therefore, it is essential to emphasize that a periodontal evaluation should be performed in patients with rosacea. Also, more studies are needed to elucidate the role of MMP-9 in the GCF of individuals with rosacea.

We performed a multiple linear regression model to elucidate whether the changes in the concentration of MMP-9 in the GCF of individuals with rosacea come from the periodontal tissue or rosacea. According to our model, rosacea (but not periodontitis, tobacco smoking, and age) could be responsible for 32% of the increased level of MMP-9 in the GCF. We hypothesize that the GCF content may be modified by the presence of unaccounted comorbidities in rosacea patients. Therefore, with these results, we provide new evidence that rosacea can change the composition of the GCF and exert functions at a distance. However, we propose to study the composition of the gingival microbiota in patients with rosacea and assess whether the presence or periodontal bacterial load influences the levels of MMP-9 in the GCF. A negative association was reported between MMP-9 and *Fusobacterium nucleatum*, *Porphyromonas gingivalis*, *Tannerella forsythia*, *Prevotella intermedia*, and *Prevotella nigrescens,* in GCF in individuals with chronic periodontitis, suggesting an anti-inflammatory effect of MMP-9 in periodontitis [58].

Considering that the GCF is a non-invasive method and readily available biomarker source for systemic disease diagnosis [26], we evaluated if the detection of MMP-9 in the GCF serves as a biomarker of rosacea. Today, no ROC curve areas are available for the tear and serum and the GCF levels of MMP-9 in rosacea diagnosis. However, we demonstrated that detecting MMP-9 in the GCF effectively diagnoses rosacea (ROC 0.8694). Previously, MMP-9 in saliva showed a 0.67 area under the ROC curve for the detection of periodontitis [59]. Therefore, MMP-9 in the GCF could be a diagnostic biomarker for rosacea. We suggest evaluating the ROC curve of MMP-9 in the GCF after rosacea treatment.

The present study presents the limitation of all observational studies where it is impossible to determine causality. Also, we cannot determine the concentration of MMP-2 in the same kit including MMP-9, in the GCF.

## 4. Materials and Methods

### 4.1. Study Design

We designed a cross-sectional research study at the Dental Clinic of the Faculty of Dentistry of Universidad Andrés Bello, Santiago, Chile. The Ethics-Scientific Committee of the Faculty of Dentistry of Universidad Andrés Bello approved the research protocol (#PROPRGFO_2022_76). The final manuscript followed the STROBE (Strengthening the Reporting of Observational Studies in Epidemiology) guidelines for reporting cross-sectional studies [60].

### 4.2. Study Participants

Dentists from the Oral Diagnosis Clinic of Universidad Andrés Bello invited systemically healthy adults with alleged rosacea to participate in the study. All participants provided written consent before enrollment, following the institutional and national ethical standards and the Helsinki Declaration for protecting human participants in medical research [61]. Experienced dermatologist of the Dermatology and Venereology Department at Hospital San José, Santiago, Chile clinical confirmed the rosacea diagnosis via teleconsultation. For this purpose, a trained operator took extraoral photographs of each patient, following the “Quality Standards for Teledermatology: Using ‘Store and Forward’ Images, Primary Care Commissioning 2013” and the “UK Guidance on the use of Mobile Photographic Devices in Dermatology. Primary Care Commissioning 2017” guidelines by the British Association of Dermatologists [62], British Society of Teledermatology tBAoDB; Scottish dermatologists, The British Dermatological Nursing Group (BDNG); The Primary Care Dermatology Society (PCDS). UK Guidance on the Use of Mobile Photographic Devices in Dermatology; The British Dermatological Nursing Group (BDNG): London, UK, 2017). The researchers used a secure smartphone application (PicSafe^®^ for Android, Melbourne, Australia, Slay Pty Ltd.) to guarantee patient confidentiality, data security, safe storing, and sharing of the images. The study’s main exclusion criteria were: (a) pregnant women, (b) subjects with non-communicable inflammatory or autoimmune diseases (i.e., diabetes, psoriasis, rheumatoid arthritis, to name a few), (c) patients with a history of treatment with antibiotics, immunosuppressant, steroidal, and non-steroidal anti-inflammatory drugs in the past three months, (d) individuals with a history of radio/chemotherapy treatment in the last year, (e) patients with less than ten functional teeth and (f) those who received periodontal treatment in the last six months. Controls met the same criteria except for the rosacea.

### 4.3. Clinical Evaluations

A qualified periodontist (C.J), with the assistance of a dentistry student, registered participants’ medical history, sociodemographic characteristics, smoking activity, and oral hygiene habits. They also made the periodontal evaluations, including full-mouth periodontal charting and recording, using a manual UNC-15 periodontal probe (UNC-15 periodontal probe, Hu-friedy^®^, Chicago, IL, USA). Periodontal clinical parameters, including clinical attachment level (CAL), probing depth (PD), and bleeding on probing (BOP), were individually measured at six sites per tooth, excluding third molars. They assessed periodontal health status and periodontitis diagnosis using the joint clinical classification system devised by Page & Eke for population-based studies [63]. All participants diagnosed with periodontitis were derived to the Periodontal Teaching Clinic at Universidad Andrés Bello for further evaluation and prompt treatment.

### 4.4. Gingival Crevicular Fluid (GCF) Sampling and Analysis

As previously described, the same periodontist (C.J) collected crevicular fluid samples at the most profound site per quadrant preventing saliva contamination. The periodontist cautiously isolated the chosen sites using sterile cotton rolls and gentle air-drying with the dental-chair air syringe system. Then, placed sterile paper strips (Periopaper^®^, Oraflow, Plainview, NY, USA), for 30 s, in the periodontal sulcus and pockets and saved them into sterile laboratory tubes (Eppendorf^®^, Eppendorf AG, Hamburg, Germany). Immediately after, they were transferred to the Periodontal Biology Laboratory at the Faculty of Dentistry of the Universidad de Chile, Santiago, Chile, for storage, testing, and analysis.

A trained lab analyst made the GCF analyses using pooled samples from each individual and prepared the specific elution by adding forty microliters of protein buffer solution to each tube. The dilutions were then incubated at 4 °C for thirty minutes and immediately centrifuged at 12,000× *g* for five minutes at the same temperature [36]. The procedure was repeated twice to optimize the isolation of proteins. Then, the analyst analyzed the aliquots from all participants using a multiplex bead immunoassay (Human Magnetic Luminex Assay^®^, R&D Systems, Minneapolis, MN, USA). In this process, the samples were diluted (1:50) using the panel kit buffer provided by the manufacturer. This kit was designed to quantify MMP2, and MMP9 concentrations. The concentrations of MMP were quantified using a digital platform (Magpix, Millipore, St. Charles, MO, USA) and later analyzed with the MILLIPLEX AnalystR software^®^ (v5.1, Viagene Tech, Carlisle, MA, USA). Nonetheless, we could not code MMP-2 levels due to less than minimum detection levels.

### 4.5. Sample Size Calculation

Previously reported serum and tears concentrations of MMP9 in patients with rosacea and healthy subjects were used to calculate the sample size requirements for this research [24,34]. The determining effect size was 0.63 and 3.74, respectively. We estimated an effect size 1.0, with a significance level of α = 0.05 and a power of 0.8. The results showed that the study needed a minimum sample size of 17 individuals per group.

### 4.6. Statistical Analysis

We performed statistical analyses using the STATA v13^®^ StataCorp software (StataCorp. LLC, College Station, TX, USA). First, we evaluated the normality of distribution and homoscedasticity using Shapiro–Wilk and Levene’s tests. We conducted inferential analyses using Student’s *t*-test and Fisher exact tests with a 0.05 significance level, using the log transformation of MMP-2 and -9 concentrations. We also used a multiple linear regression model controlling for periodontitis, age, and tobacco use. Finally, to evaluate the performance discrimination and diagnostic precision of studied molecules, we used receiver operating characteristic (ROC) curves and estimated the area under the curve (AUC) for the rosacea and control groups.

## 5. Conclusions

The GCF levels of MMP-9 were different between the rosacea and healthy control group. rosacea was responsible for increasing the levels of MMP-9 in the GCF independent of periodontal status. Also, detection of MMP-9 in GCF may be used as biomarker for rosacea diagnosis.

## Figures and Tables

**Table 1:** Detailed demographic parameters and periodontal evaluation in rosacea and Control groups.

Parameters	R (*n* = 18)	C (*n* = 20)	*p*
Age: years (mean ± SE)	33.05 ± 9.92	35.3 ± 13.98	0.576
Female (%-*n*) male (%-*n*)	94.44-17 5.56-1	65.0-13 35.0-7	**0.045**
Smokers (%-*n*)	50-9	20-4	0.087
PD (mean ± SE)	1.90 ± 0.32	1.83 ± 0.35	0.538
CAL (mean ± SE)	1.65 ± 0.67	1.38 ± 0.31	0.134
BOP (mean ± SE)	7.21 ± 7.33	7.98 ± 10.00	0.789
Periodontitis Mild (%) Moderate (%) Severe (%)	16.67 44.44 16.67	20 35 0	0.181

PD: Probing depth; CAL: Clinical attachment level, BOP: bleeding on probing; SE: Standard error, bold: *p* < 0.05.

**Table 2:** Results of de multiple regression models for MMP-9 levels in gingival crevicular fluid in rosacea and control patients.

*n* = 38	MMP-9 Levels		
Variables	Coef. ± SE	t	*p*
Diagnosis of rosacea	0.925 ± 0.269	3.44	**0.002**
Mild periodontitis	0.412 ± 0.356	1.16	0.257
Moderate periodontitis	0.154 ± 0.336	0.46	0.649
Severe periodontitis	0.695 ± 0.553	1.26	0.219
Age (years)	−0.002 ± 0.012	−0.16	0.877
Smoker	−0.008 ± 0.281	−0.03	0.977
Gender	−0.008 ± 0.363	−0.02	0.982
β	5.293 ± 0.443	11.94	0.000
Adj. R^2^	0.300		

Coef: coefficient, SE: Standard error, bold: *p* < 0.005.

## Data Availability

Not applicable.
